# A Case of Long-Term Survival after Multimodal Treatments including Living-Donor Liver Transplantation for a Primary Neuroendocrine Tumor of the Bile Duct

**DOI:** 10.70352/scrj.cr.26-0255

**Published:** 2026-08-27

**Authors:** Akihito Jo, Akira Umemura, Hiroyuki Nitta, Satoshi Amano, Taro Ando, Toma Kawashima, Koji Kikuchi, Taku Kimura, Ayaka Sato, Naoki Yanagawa, Akira Sasaki

**Affiliations:** 1 Department of Surgery, School of Medicine, Iwate Medical University, Shiwa-gun, Iwate, Japan; 2 Department of Molecular Diagnostic Pathology, School of Medicine, Iwate Medical University, Shiwa-gun, Iwate, Japan

**Keywords:** neuroendocrine tumor, extrahepatic biliary neuroendocrine tumor, liver transplantation, liver metastases

## Abstract

**INTRODUCTION:**

An extrahepatic biliary neuroendocrine tumor (EB-NET) is an extremely rare malignancy with a poor prognosis and no established standard of care.

**CASE PRESENTATION:**

We herein report the case of a 35-year-old Japanese woman with no significant past medical or family history who presented with epigastric and back pain and was diagnosed with an EB-NET after pylorus-preserving pancreaticoduodenectomy. Despite initial resection, she developed recurrent liver metastases that required repeated hepatic resections and multiple systemic therapies, including chemotherapy, somatostatin analogs, everolimus, and peptide receptor radionuclide therapy (PRRT). Eventually, owing to progressive liver metastasis, she underwent living-donor liver transplantation (LDLT) from her brother 6 years after the initial surgery. Post-transplantation, the patient has remained disease-free for >3 years while on maintenance immunosuppression.

**CONCLUSIONS:**

Multimodal treatment, including resection of metastases, PRRT, and LDLT, significantly prolonged survival. This case highlights the potential role of LDLT as a curative strategy for patients with EB-NETs and controlled extrahepatic disease following multimodal treatment. Our case suggests that liver transplantation may provide long-term survival even in cases of a rare primary EB-NET, prompting the consideration of broader transplant indications in selected patients.

## Abbreviations

CA19-9
: carbohydrate antigen 19-9
CA125
: cancer-associated antigen 125
CEA
: carcinoembryonic antigen
EB-NET
: extrahepatic biliary neuroendocrine tumor
LDLT
: living-donor liver transplantation
NET
: neuroendocrine tumor
NSE
: neuron-specific enolase
PPPD
: pylorus-preserving pancreaticoduodenectomy
PRRT
: peptide receptor radionuclide therapy

## INTRODUCTION

The incidence of pancreatic and gastrointestinal NETs is increasing.^1)^ However, EB-NETs are extremely rare, accounting for only 0.2%–2.0% of all NETs.^2)^ Because of this low incidence, there are no standardized guidelines for diagnosis or treatment, and the prognosis is considered poor.^3)^

Treatment options for NETs include pharmacologic therapy, nuclear medicine approaches such as PRRT, and surgical resection, with liver transplantation also considered in selected cases.^4)^

Here, we report an extremely rare case of an EB-NET in a 35-year-old woman who underwent multimodal treatment, including initial resection, repeated hepatic resections, and ultimately LDLT, achieving long-term survival. This case suggests that multidisciplinary treatment, including liver transplantation, may be necessary to improve outcomes in patients with recurrent hepatic metastases.

## CASE PRESENTATION

A 35-year-old Japanese woman presented 9 years ago with epigastric and back pain. She was referred to our hospital for further evaluation because a biliary tumor was suspected. She had no family history of malignancy or endocrine disease and no significant medical history. She did not smoke and reported moderate alcohol intake (approximately 350 mL of beer 3 times per week and 1 glass of wine per day). Laboratory findings showed normal liver function: aspartate aminotransferase, 31 IU/L; alanine aminotransferase, 18 IU/L; and total bilirubin, 0.7 mg/dL. The tumor marker CA125 was elevated at 55.3 U/mL, whereas CEA was 2.0 ng/mL, CA19-9 was 0.6 U/mL, and NSE was 9.7 ng/mL, which were within normal limits.

**Fig. 1:**
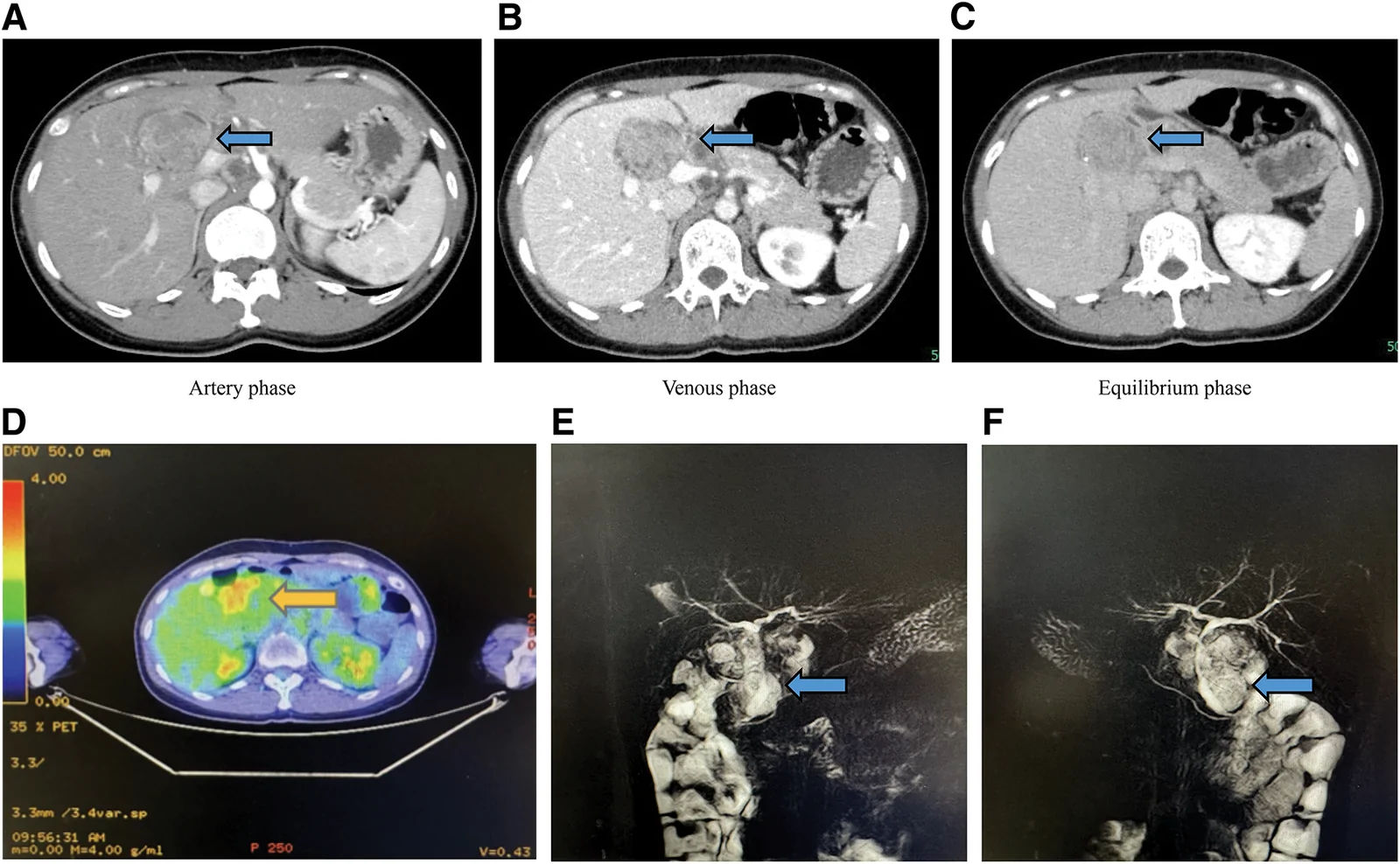
(**A**–**C**) A mixed cystic and solid lesion extending from the pancreatic head along the hepatoduodenal ligament. The solid component shows contrast enhancement (arrow). (**D**) Abnormal fluorodeoxyglucose uptake (arrow) with a maximum standardized uptake value of 3.8. (**E**, **F**) A mass (arrow) compressing the common bile duct from both anterior and posterior sides.

**Fig. 2:**
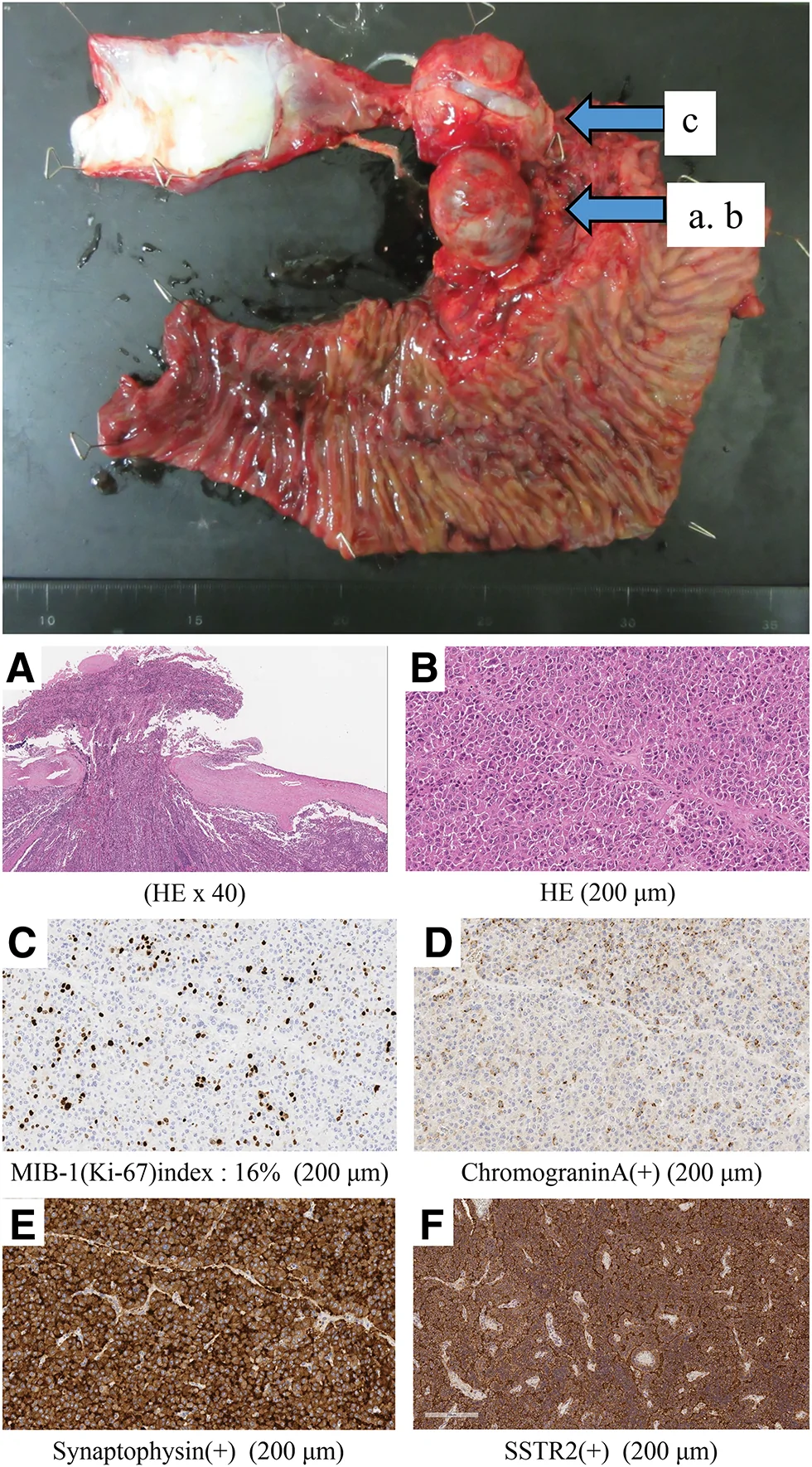
(**A**–**C**) Three nodular lesions (a-c; arrows) along the bile duct and cystic duct around the confluence of the hepatic ducts. (**A**, **B**) Tumor cells with granular ovoid nuclei forming trabecular, reticular, and ribbon-like solid nests separated by highly vascularized stroma. No continuity with the biliary or cystic duct epithelium is observed. (**C**–**F**) The MIB-1 index is 16%, and immunostaining is positive for chromogranin A, synaptophysin, and SSTR. HE, hematoxylin and eosin; SSTR, somatostatin receptor

A gastrointestinal examination revealed no abnormalities. Contrast-enhanced CT demonstrated a 44-mm mass with cystic and solid components in the pancreatic head extending along the hepatoduodenal ligament, with early arterial-phase enhancement (**Fig. 1A**–**1C**). PET/CT showed abnormal uptake in the same lesion (**Fig. 1D**). Magnetic resonance cholangiopancreatography revealed preserved structures of the bile and pancreatic ducts (**Fig. 1E** and **1F**). Although a definitive radiologic diagnosis could not be established, the differential diagnoses included EB-NET and intraductal papillary neoplasm of the bile duct, leading to the decision to perform PPPD (**Fig. 2**).

Histopathological examination revealed 3 nodular lesions along the biliary tract and cystic duct in the hepatoduodenal ligament, measuring 18 × 13 mm (a), 20 × 20 mm (b), and 38 × 30 mm (c), respectively (**Fig. 2**). The largest lesion contained tumor cells with granular, oval nuclei forming trabecular, reticular, and ribbon-like solid nests beneath the biliary epithelium (**Fig. 2A**). The other 2 nodules represented metastatic lymph nodes (**Fig. 2B**). Immunohistochemistry showed a Ki-67 index of 16% (**Fig. 2C**), chromogranin A positivity (**Fig. 2D**), and synaptophysin positivity (**Fig. 2E**), consistent with NET G2. A selective silver stain for reticulin 2 on the cell membrane was also positive (**Fig. 2F**). After surgery, the CA125 level, which was initially elevated to 55.3, decreased promptly to 32.5 and subsequently to 21.7, returning to within the normal range 2 months later, with no further elevation observed thereafter.

**Fig. 3:**
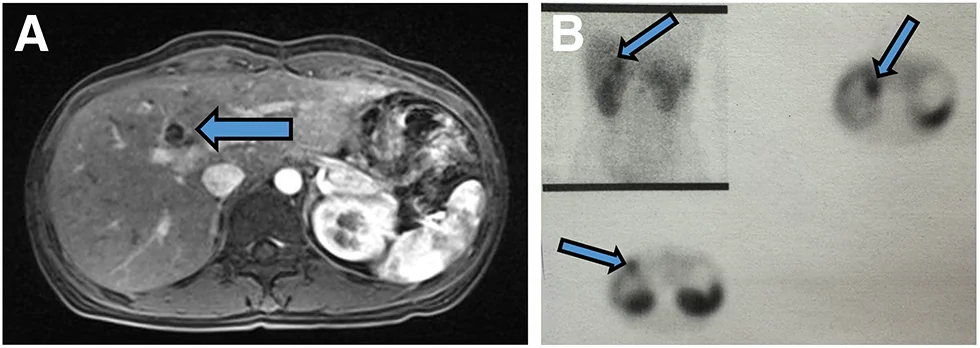
(**A**) A hepatic mass (arrow) suspected of metastasis in segment 4. (**B**) Abnormal accumulation (arrow) in the same region on PET.

**Fig. 4:**
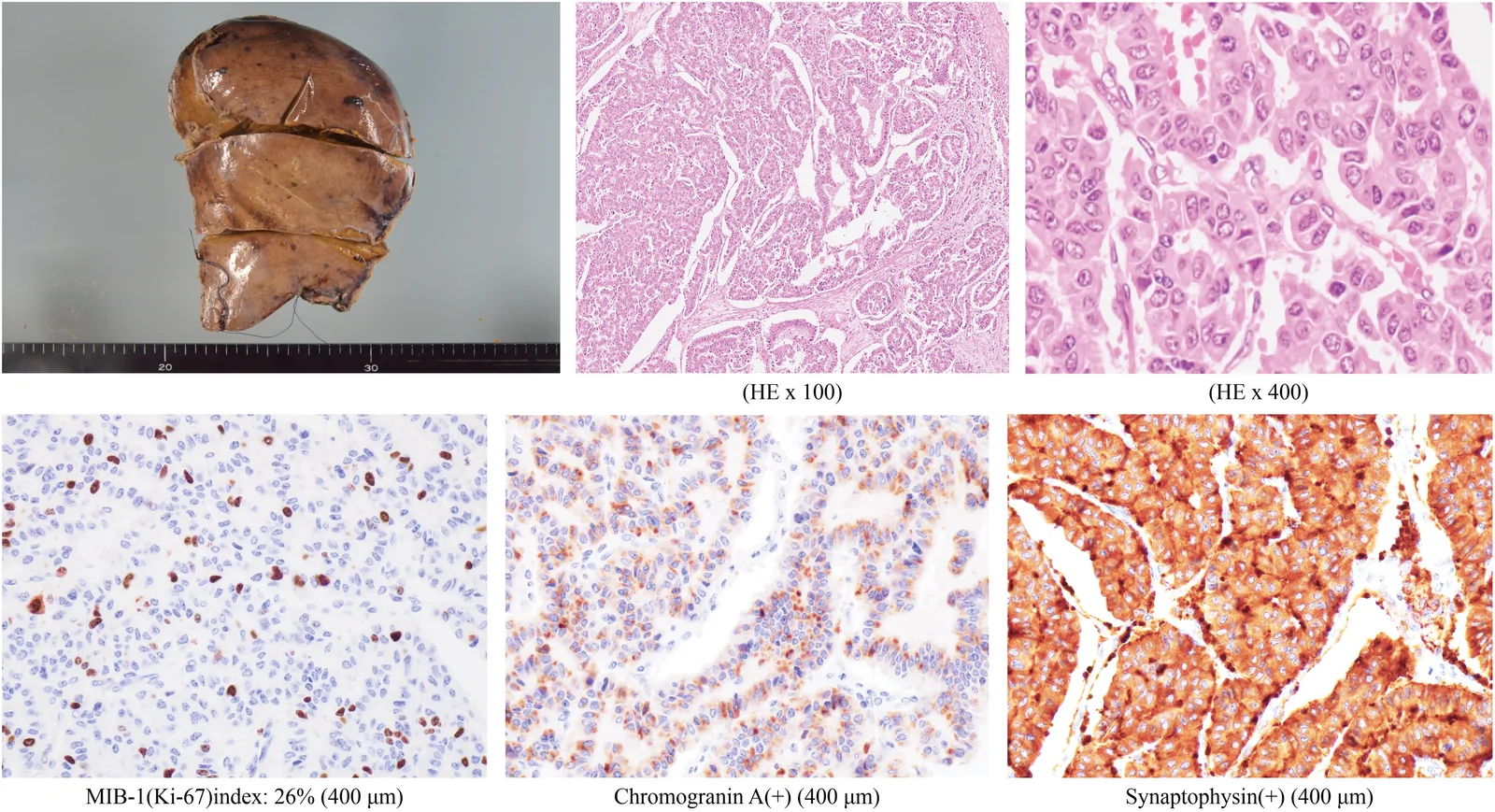
Histological findings similar to those in **Fig. 2**, with an increased MIB-1 index of 26%. HE, hematoxylin and eosin

No elevation of CEA, CA19-9, or NSE was observed. Postoperatively, the patient received 2 cycles of adjuvant chemotherapy with irinotecan and cisplatin, which were discontinued because of adverse events. She was placed under close follow-up because of her young age and the early discontinuation of therapy. One year and 4 months after the initial surgery, liver metastases in segments 4, 5, and 8 were detected by MRI and an octreotide scan (**Fig. 3A** and **3B**), prompting an extended right anterior sectionectomy and partial S1 resection. Histopathological analysis of the liver metastases showed features consistent with the initial tumor but with a higher Ki-67 index of 26%, meeting the criteria for NET G3 (**Fig. 4**) and indicating a histological upgrade from the original NET G2 classification. Detection of gastrin-positive cells suggested a potentially functional tumor, for which somatostatin analog therapy was considered.

**Fig. 5:**
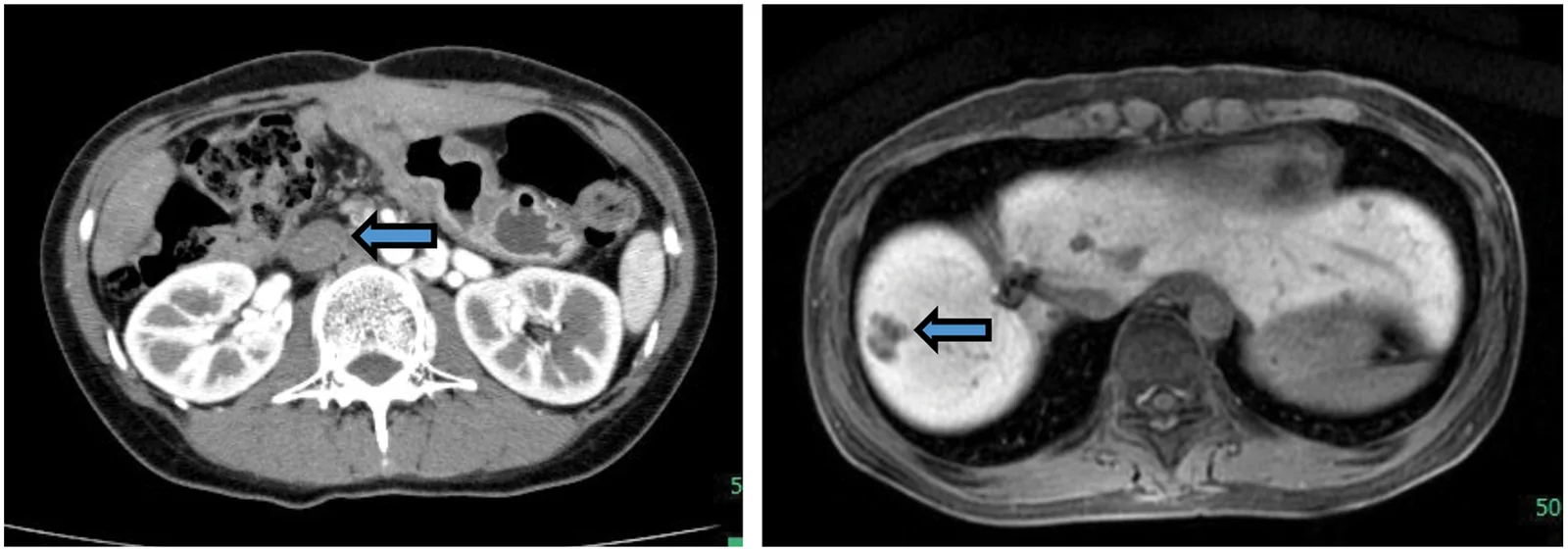
CT showing para-aortic lymphadenopathy and MRI revealing hepatic metastases.

After liver resection, the patient began subcutaneous lanreotide acetate because of the positive histopathological findings for somatostatin receptor subtype 2. Recurrent lesions in the remnant liver were suspected on MRI performed 1 year and 10 months after the initial surgery, prompting the initiation of everolimus therapy. Two years and 10 months after the initial surgery, CT and MRI revealed progression of hepatic and para-aortic lymph node metastases, leading to a switch from everolimus to sunitinib and to temozolomide plus capecitabine (**Fig. 5**). Despite these treatments, the liver metastases continued to increase in size, with the lesion enlarging from 13 to 18 mm.

**Fig. 6:**
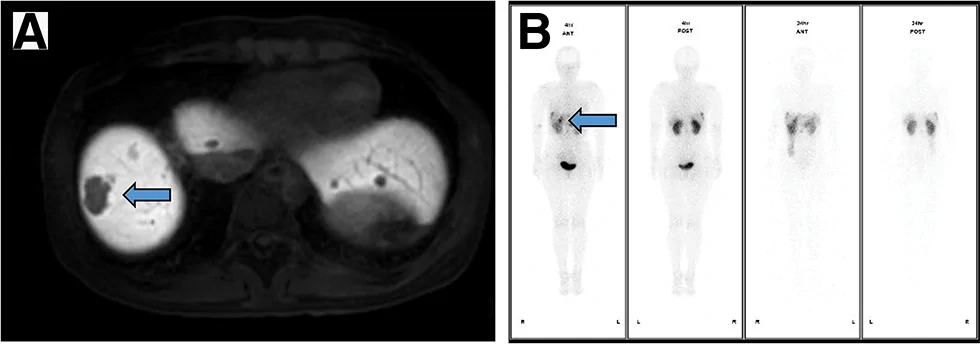
(**A**) Enlargement of hepatic metastases. (**B**) Corresponding abnormal uptake on octreotide scintigraphy.

**Fig. 7:**
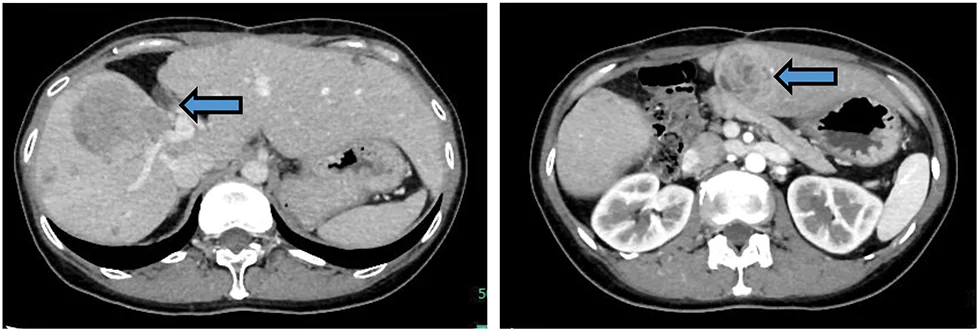
Progressive enlargement of multiple hepatic metastases in both lobes.

Therefore, PRRT was administered along with continued lanreotide therapy. The disease remained stable for 2 years after PRRT, but subsequent MRI and octreotide scans showed renewed progression (**Fig. 6A** and **6B**). The patient continued lanreotide acetate and experienced temporary stability; however, despite resection of the primary lesion, PRRT, and repeated hepatectomy, liver metastases continued to recur and progress. Although disease stabilization for more than 6 months had not been achieved, LDLT was exceptionally considered as a last-resort therapeutic option under self-funded medical care. Seven years after the initial surgery, CT revealed further progression of liver metastases, leading to the decision to perform LDLT (**Fig. 7**). The donor was her ABO-compatible brother. The donor liver volume was 1500 mL (right lobe 72.3%, left lobe 27.7%). The graft-to-recipient weight ratio was 0.83, and the graft volume/standard liver volume was 39%. LDLT was performed using a left-lobe graft without the caudate lobe, with cold and warm ischemia times of 29 and 32 min, respectively. Hepatic arterial reconstruction was performed by anastomosis between the recipient right hepatic artery and the graft left hepatic artery, and biliary reconstruction was performed by anastomosis between the left hepatic duct and the jejunum. The para-aortic lymph node identified on preoperative CT was also dissected concomitantly. The operative time was 636 min, and the estimated blood loss was 545 mL.

Intraoperative donor liver biopsy showed no steatosis, fibrosis, or inflammatory infiltration. The explanted recipient liver contained 2 solid tumors measuring 85 × 30 mm and 35 × 22 mm, respectively. Histologically, these tumors consisted of nests of atypical neuroendocrine cells with granular nuclei forming ribbon-like and solid structures, and the diagnosis remained NET G3, consistent with prior pathology. Para-aortic lymph node metastasis was also confirmed.

**Fig. 8:**
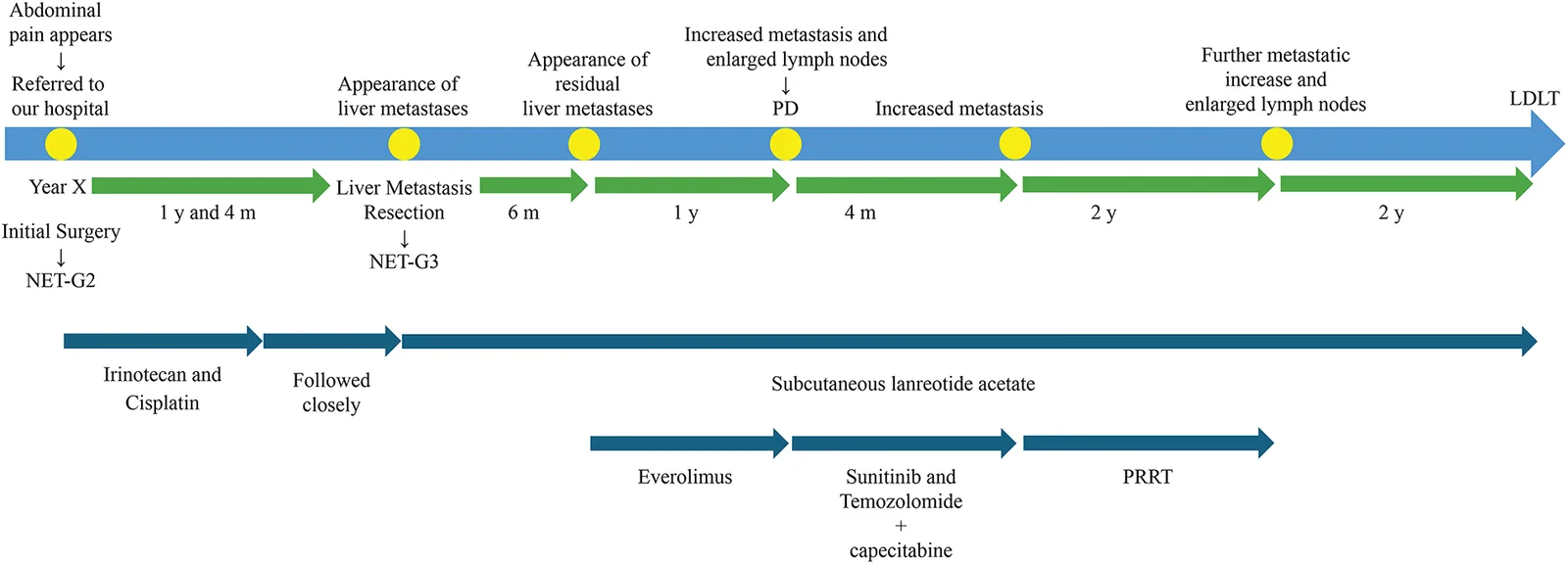
Timeline of the present case. LDLT, living-donor liver transplantation; NET, neuroendocrine tumor; PRPT, peptide receptor radionuclide therapy

The patient was discharged on POD 24 after LDLT. Immunosuppressive therapy with everolimus (1 mg/day) was initiated 1 month after surgery (**Fig. 8**). The patient has remained recurrence-free for more than 1 year after discharge.

## DISCUSSION

We describe a rare case of an EB-NET in a 35-year-old woman who achieved long-term survival after a stepwise, multimodal strategy: PPPD, repeated hepatic resections, multiple systemic regimens (including somatostatin analogs and everolimus), and PRRT, followed by LDLT for progressive liver-dominant disease. Under maintenance immunosuppression, the patient has remained disease-free, suggesting that with careful selection and prior disease control using multimodal therapy, transplantation may be curative.

Treatment options for EB-NET include surgical resection, chemotherapy, and PRRT.^5)^ However, hepatic metastases frequently develop after initial surgery, and despite additional interventions such as hepatic resection or transarterial chemoembolization, recurrence is common.^6)^ In such cases, liver transplantation may be considered a curative option.^7,8)^

In Japan, 19 cases of LDLT for metastatic liver tumors have been reported, including 16 cases involving NET.^7)^ The 5-year overall survival rate among the 16 patients who underwent LDLT in Japan was 50%.^7)^ However, most of these cases involved hepatic metastases originating from gastrointestinal or pancreatic NETs, and reports of liver transplantation for EB-NET remain extremely rare. A PubMed search identified only 2 prior cases of liver transplantation for EB-NET aside from our case (**Table 1**).

**Table 1:** Summary of the present case and previously reported cases of liver transplantation for NETs

Author/Year	Age	Sex	Primary site	Diagnostic method	Preoperative diagnosis	Liver metastasis	MELD score	Child pugh	Ki-67, %	Transplantation type
–	42	F	Junction of bile Ducts	Surgery	NET-G3	+	6	5	26	LDLT
Moradi^9)^/2023	37	F	Proximal common bile duct	EUS	NET-G1	–	8	5	2	DDLT
Rai^10)^/2023	9	F	Hepatic bile duct	Liver biopsy	NET-G1	–	–	–	<2	DDLT

DDLT, deceased-donor liver transplant; EUS, endoscopic US; F, female; LDLT, living-donor liver transplantation; NET, neuroendocrine tumor

The first case involved a 37-year-old woman with a primary NET of the proximal common bile duct complicated by chronic biliary obstruction and liver fibrosis. Because of the extent of fibrosis and tumor spread, surgical resection was not feasible, and deceased-donor liver transplantation was performed.^9)^ The second case involved a 9-year-old girl with extensive bilateral hepatic metastases from an EB-NET, who also underwent deceased-donor liver transplantation.^10)^ Both patients achieved long-term survival of more than 4 years without recurrence. Compared with these cases, our patient underwent LDLT from a living donor, which enabled timely transplantation and may have contributed to favorable outcomes. Another notable difference is that the Ki-67 index was low (approximately 2%) in the previous cases, whereas in our patient, it was elevated to 26%, meeting the criteria for NET G3.

The Milan selection criteria were proposed as indication criteria for liver transplantation in patients with liver metastases from gastroenteropancreatic NET.^11)^ According to these criteria, liver transplantation may be considered when the tumor is histologically confirmed as low grade (G1 or G2), the primary tumor is drained by the portal venous system and has been completely resected, and all extrahepatic tumor deposits have been removed with curative intent before transplant consideration. Additional criteria include metastatic involvement of less than 50% of the total liver volume and stable disease or response to therapy for at least 6 months before transplantation. Age younger than 60 years is considered a relative criterion.^11)^ On the other hand, in the 37-year-old case, liver transplantation was performed without prior resection of the primary tumor, while in the 9-year-old case, transplantation was performed without achieving long-term disease stabilization. In the present case as well, liver transplantation was performed despite the high-grade nature of the tumor and the absence of disease stabilization. In addition, para-aortic lymph node dissection performed at the time of liver transplantation revealed metastatic involvement. Nevertheless, long-term survival was achieved in all cases. These findings suggest that, in NETs originating from the bile duct, the indications for liver transplantation may potentially be expanded beyond the Milan criteria with respect to tumor grade and disease status.

Although liver transplantation for metastatic gastrointestinal and pancreatic NETs has been reported with favorable outcomes, no strict consensus guidelines exist for its indications.^9)^ Mazzaferro et al. demonstrated that the 5-year survival rate for patients with unresectable NETs who underwent liver transplantation was 97.2%, compared with 50.9% in those treated without transplantation.^11)^ Proposed criteria for liver transplantation in NETs include low tumor grade (G1 or G2), hepatic tumor burden of <50% of the liver volume, and stable disease for at least 6 months following multimodal therapy.^11)^

Although our patient had a Ki-67 index of 26%, corresponding to NET G3, her hepatic tumor burden was <50%, and she had undergone multiple prior resections and therapies. This case suggests that liver transplantation may still offer long-term survival, even in carefully selected patients with higher tumor grades. Moreover, it is necessary to evaluate extrahepatic disease and to achieve disease control before considering transplantation. After PRRT, disease stability was achieved for approximately 2 years. In retrospect, liver transplantation might have been feasible during this period. However, during the period of disease stability, we did not proceed with liver transplantation because of the high-grade tumor biology of the tumor and the clinical context following extensive hepatic resection. In the present case, liver transplantation was ultimately performed as a last-resort treatment after disease stabilization could no longer be achieved.

Currently, only 3 cases—including our patient—have undergone liver transplantation for EB-NETs, all achieving long-term survival. Although liver transplantation is recognized as a potentially curative treatment for patients with hepatic metastases from pancreatic and gastrointestinal NETs, strict application of current transplantation criteria may limit its use in EB-NETs. To establish appropriate selection criteria for liver transplantation in patients with EB-NET and liver metastases, large-scale prospective studies will be necessary in the future. It would be useful to conduct a multicenter study collecting cases of EB-NET with liver metastases treated with multimodal therapy and comparing patients who underwent liver transplantation with those who did not. The primary endpoints should include overall survival and recurrence-free survival. Secondary endpoints could include tumor grade at the time of transplantation, Ki-67 index, hepatic tumor burden, disease stability before transplantation, and the types of treatments performed before transplantation, such as surgical resection, systemic therapy, PRRT, or locoregional therapy. Such an approach may help clarify the prognostic factors associated with favorable outcomes and could contribute to the potential expansion of indications for liver transplantation in selected patients with EB-NET.

## CONCLUSIONS

We presented a rare case of an EB-NET treated with multimodal therapy, including initial surgical resection, repeated hepatic resections, multiple systemic therapies, and ultimately LDLT, achieving long-term survival. This case demonstrates that LDLT may be a potential curative option for patients with recurrent hepatic metastases from EB-NETs.

Our experience suggests that the current criteria for liver transplantation may need to be reconsidered and broadened to include carefully selected cases of EB-NET. Additional clinical cases and large-scale prospective studies are needed to establish clearer guidelines for the indication of liver transplantation in this rare disease.

## DECLARATIONS

### Availability of data and materials

All data generated or analyzed during this study are included in this published article.
